# Supplementary material for: Efficacy and safety of a new ranibizumab biosimilar CKD-701 using a pro re nata treatment regimen in neovascular age-related macular degeneration: A phase 3 randomized clinical trial
Source: PLoS One. 2022 Nov 14;17(11):e0275611. doi: 10.1371/journal.pone.0275611 (PMC9662729; doi:10.1371/journal.pone.0275611)
Supplement: S1 File — (PDF) [file pone.0275611.s002.pdf]

A Multicenter, Randomized, Double-blind, Active-controlled, Parallel group, Phase III Clinical Trial to Evaluate the Efficacy, Safety, Pharmacokinetics and Immunogenicity of CKD-701 and Lucentis® in Patients with Neovascular(wet) Age related Macular Degeneration

PROTOCOL VERSION 5.2
25 Jan 2021

Chong Kun Dang Pharmaceutical Corp.

Abbreviations and Terms

Test Drug	CKD-701 (generic name: ranibizumab), Chong Kun Dang Pharmaceutical Corp.
Comparator	Lucentis® (generic name: ranibizumab), Novartis Korea
Investigational Product	Test drug, comparator
ADA	Anti-drug Antibody
ADR	Adverse Drug Reaction
AE	Adverse Event
AESI	Adverse Events of Special Interest
AMD	Age-related Macular Degeneration
BCVA	Best Corrected Visual Acuity
CDMS	Clinical Data Management System
CRT	Central Retinal Thickness
CNV	Choroidal Neovascularization
DA	Disc Area
ETDRS Chart	Early Treatment Diabetic Retinopathy Study Chart
FA	Fluorescein Angiography
FAS	Full Analysis Set
ICGA	Indocyanine Green Angiography
IOP	Intraocular Pressure
IP	Investigational Product
IRB	Institutional Review Board
MedDRA	Medical Dictionary for Regulatory Activities
OCT	Optical Coherence Tomography
PCV	Polypoidal Choroidal Vasculopathy
PK	Pharmacokinetics
PKS	Pharmacokinetic analysis Set
PPS	Per-protocol Set
SAE	Serious Adverse Event
TEAE	Treatment Emergent Adverse Event
VEGF	Vascular Endothelial Growth Factor

1. Title

A Multicenter, Randomized, Double-blind, Active-controlled, Parallel group, Phase III Clinical Trial to Evaluate the Efficacy, Safety, Pharmacokinetics and Immunogenicity of CKD-701 and Lucentis® in Patients with Neovascular(wet) Age related Macular Degeneration

2. Objectives

CKD-701 or Lucentis® will be administered to patients with neovascular (wet) age-related macular degeneration (AMD) as an intravitreal injection,

1) Primary Objective: To demonstrate that the efficacy of CKD-701 is equivalent to that of Lucentis®.
2) Secondary Objectives
   - To evaluate the safety of CKD-701 and Lucentis®.
   - To evaluate the pharmacokinetics (PK) of CKD-701 and Lucentis®.
   - To evaluate the immunogenicity of CKD-701 and Lucentis®.

3. Target Disease

Neovascular (wet) age-related macular degeneration (AMD)

4. Subjects

In this study, only one eye is selected as the study eye for evaluation. If only one eye of the subject meets the inclusion criteria, that eye becomes the study eye; if both eyes meet the inclusion criteria, the eye most recently diagnosed with neovascular (wet) AMD is selected as the study eye. However, depending on the investigator's judgment, the study eye can be selected based on other criteria; and the reason should be recorded in this case.

[Inclusion Criteria]

- ≥50 years of age
- [Study eye] Active subfoveal choroidal neovascularization (CNV) lesion secondary to AMD

- [Study eye] Total lesion size ≤12 DA, including blood, scars, and neovascularization at screening
- [Study eye] CNV must be at least 50% of total lesion size at screening
- [Study eye] BCVA of 20/32 to 20/200 (letter score of 78 to 34, ETDRS) at screening and randomization (Visit 2)
- Patients who voluntarily decided to participate in this study and signed the informed consent form

[Exclusion Criteria]

- [Study eye or fellow eye] Prior treatment with intravitreal anti-vascular endothelial growth factor (anti-VEGF) drugs for the treatment of AMD (bevacizumab, aflibercept, ranibizumab, pegaptanib, anecortave acetate, protein kinase C (PKC) inhibitors). For the fellow eye, if the anti-VEGF drug was administered 60 days prior to the randomization date, the patients can participate
- [Study eye or fellow eye] Any intraocular, extraocular, or periocular inflammation or infection within 30 days prior to randomization, including infectious blepharitis, keratitis, scleritis (including scleromalacia), and endophthalmitis
- [Study eye or fellow eye] Prior or current idiopathic or autoimmune uveitis at screening
- [Study eye or fellow eye] History or clinical evidence of diabetic retinopathy, diabetic macular edema, or any other diseases affecting the retina, other than AMD (Patients with non-proliferative diabetic retinopathy can participate at the investigator's discretion)
- [Study eye] CNV due to other reasons, such as ocular histoplasmosis, trauma, angioid streaks retinopathy, and pathologic myopia

5. Number of Subjects

310 in total (155 per group, dropout rate of 15%)

6. Study Design

A Multicenter, Randomized, Double-blind, Active-controlled, Parallel group, Phase III Clinical Trial

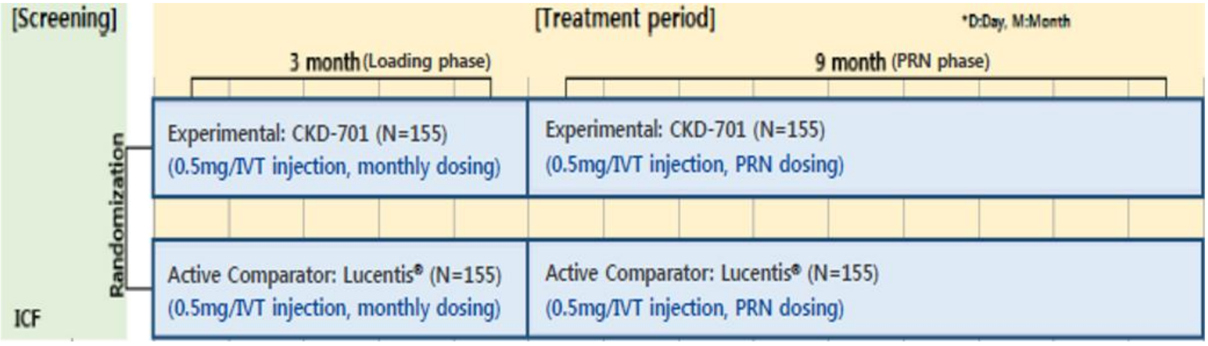

The study procedures begin after obtaining the voluntary informed consent form from the subject, and a total of 15 site visits are conducted, including a screening visit (Visit 1). However, the 24 subjects who undergo PK blood sampling should visit the site for a total of 16 visits, including Visit 2-1. The inclusion/exclusion criteria are assessed at the screening visit (Visit 1), and at Visit 2 (Day 0), the IP administration begins after the final evaluation of the inclusion/exclusion criteria, selection of the study eye, and randomization. The safety is assessed at Visit 3 (Day 7), and subjects visit the site at 30-day intervals from Day 0 between Visit 4 to Visit 15. During the first 3 months (loading phase), the test drug or the comparator is administered once a month according to the randomized group, and for the following 9 months, the administration is determined according to the pre-determined dosage criteria.

7. Study Period

Four years from the date of approval from the Ministry of Food and Drug Safety (MFDS)

8. Investigational Product

1) Test drug

① Code name: CKD-701 inj. (ranibizumab 3 mg/0.3 mL)

② Manufacturer: Chong Kun Dang Pharmaceutical Corp.

2) Comparator

① Trade name (generic name): Lucentis inj. (ranibizumab 3 mg/0.3 mL)

② Manufacturer: Novartis Korea

9. Dosage and Administration

Subjects who are confirmed to be appropriate under inclusion/exclusion criteria are randomized to either the test group or the comparator group at a 1:1 ratio. During the

first 3 months, 0.5 mg (0.05 mL) of the IP is given as an intravitreal injection once a month according to the randomized group. For the following 9 months, whether or not to perform administration is determined monthly according to the pre-determined PRN dosing criteria. Administration is allowed for other reasons at the investigators' discretion, but a legitimate reason must be recorded. Subsequent administration is not permitted within 14 days of the last dose, and missed doses cannot be made up. Each vial must be used for a single intravitreal injection only, and intravitreal injection of the IP must be performed by an ophthalmologist (an investigator exclusively responsible for injections) who is qualified and has intravitreal injection experience. The person in charge of administering the IP should be designated, and the person must strictly follow the sterilization procedure throughout the preparation and administration process. After the IP is used, it should be discarded.

[PRN dosing criteria]

1) Visual acuity loss of 5 or more letters (Early Treatment Diabetic Retinopathy Study Chart, ETDRS) compared to the best visual acuity at baseline and during the study.
2) Sub-retinal fluid or intra-retinal fluid (including new or remaining fluid)
3) Sub-retinal hemorrhage or intra-retinal hemorrhage (including new or remaining hemorrhage)
4) Formation of new neovascularization
5) Center retinal thickness (CRT) increased by ≥50 μm as measured by optical coherence tomography (OCT) compared to the previous lowest value

The IP is re-administered if the above PRN dosing criteria are met after 3 months of treatment (loading phase). However, if the subject meets Dropout Criteria, IP administration is suspended; if the investigator determines that continuous administration after suspension is inappropriate, then the investigator may decide to withdraw the subject.

10. Study Method

This clinical study consists of a Screening Period and a 12-month Treatment Period.

1) Screening Period

①Visit 1 (Screening): Day -21 to Day -1

- Informed Consent is signed, Screening Number (SN) is assigned, demographic information, medical and treatment history, vital signs (blood pressure, pulse, body temperature), physical examination (head and neck, heart, lungs, abdomen, liver, skin, limbs, etc.), physical measurements (height and weight), pregnancy test (only for women of childbearing potential), ocular assessment* (BCVA, IOP measurement, OCT, fluorescein angiography (FA), indocyanine green angiography (ICGA), ophthalmoscopy (indirect ophthalmoscopy), slit lamp examination, and fundus photography), immunogenicity test, laboratory test (complete blood cell count, blood chemistry test and urinalysis), identification of previous/concomitant drugs, and evaluation of inclusion/exclusion criteria

**During the ocular assessment, BCVA is measured at a viewing distance of 1 meter when the subject reads less than 20 letters or numbers correctly at a viewing distance of 4 meters.*

However, when visual acuity is measured again at a viewing distance of 1 meter, if the number of correctly read letters or numbers is 0, then the following tests are to be performed:

: finger count, hand motion, light perception, no light perception

The above tests are performed in the same manner during the treatment period.

2) Treatment Period

①Visit 2 (Randomization): Day 0

- After the ophthalmology visit (eyesight test), a final evaluation of the exclusion and inclusion criteria is performed, the study eye is selected, followed by randomization and assignment of the Allocation Number (AN), vital signs (blood pressure, pulse, body temperature), pregnancy test (if necessary, only for women of childbearing potential), ophthalmic examination (BCVA, IOP measurement, OCT, ophthalmoscopy (indirect ophthalmoscopy), slit lamp exam, fundus photography), confirmation of concomitant medications and medical treatments, PK blood sampling (pre-dose, only for subjects requiring blood sampling for PK evaluation), IP administration, adverse event (AE) evaluation, follow-up contact

②Visit 2-1 (Treatment Period): Day 1 (Only for subjects requiring PK blood sampling)

- Vital signs (blood pressure, pulse, body temperature), PK blood sampling (Day 1, only for subjects requiring PK blood sampling), check concomitant medication and therapy, AE evaluation

③Visit 3 (Treatment Period): Day 7

- Vital signs (blood pressure, pulse, body temperature), pregnancy test (if necessary, only for women of childbearing potential), ophthalmic examination (BCVA, IOP measurement, OCT, ophthalmoscopy (indirect ophthalmoscopy), slit lamp exam, fundus photography), confirmation of concomitant medications and medical treatments, PK blood sampling (Day 7, only for subjects requiring blood sampling for PK evaluation), confirmation of concomitant medications and medical treatments, AE evaluation

④Visits 4 and 5 (Treatment Period): 1, 2 months

- Vital signs (blood pressure, pulse, body temperature), pregnancy test (if necessary, only for women of childbearing potential), ophthalmic examination (BCVA, IOP measurement, OCT, ophthalmoscopy (indirect ophthalmoscopy), slit lamp exam, fundus photography), immunogenicity test, confirmation of concomitant medications and medical treatments, PK blood sampling (Day 30, Day 60, only for subjects requiring blood sampling for PK evaluation), IP administration, AE evaluation, follow-up contact

⑤Visit 6 (Treatment Period): 3 months

- Vital signs (blood pressure, pulse, body temperature), pregnancy test (if necessary, only for women of childbearing potential), ophthalmic examination (BCVA, IOP measurement, OCT, FA, ICGA, ophthalmoscopy (indirect ophthalmoscopy), slit lamp exam, fundus photography), immunogenicity test, confirmation of concomitant medications and medical treatments, PK blood sampling (Day 90, only for subjects requiring blood sampling for PK evaluation), IP administration according to PRN dosing criteria, AE evaluation, follow-up contact (for those who had the IP administered)

⑥Visits 7 and 8 (Treatment Period): 4, 5 months

- Vital signs (blood pressure, pulse, body temperature), pregnancy test (if necessary, only for women of childbearing potential), ophthalmic examination (BCVA, IOP measurement, OCT, ophthalmoscopy (indirect ophthalmoscopy), slit lamp exam, fundus photography), confirmation of concomitant medications and medical

treatments, IP administration according to PRN dosing criteria, AE evaluation, follow-up contact (for those who had the IP administered)

⑦Visit 9 (Treatment Period): 6 months

- Vital signs (blood pressure, pulse, body temperature), pregnancy test (if necessary, only for women of childbearing potential), ophthalmic examination (BCVA, IOP measurement, OCT, FA, ICGA (conduct the tests if deemed necessary by the investigator), ophthalmoscopy (indirect ophthalmoscopy), slit lamp exam, fundus photography), immunogenicity test, laboratory tests (general blood test, blood chemistry test, urine test), confirmation of concomitant medications and medical treatments, IP administration according to PRN dosing criteria, AE evaluation, follow-up contact (for those who had the IP administered)

⑧Visits 10, 11, 12, 13, and 14 (Treatment Period): 7, 8, 9, 10, 11 months

- Vital signs (blood pressure, pulse, body temperature), pregnancy test (if necessary, only for women of childbearing potential), ophthalmic examination* (BCVA, IOP measurement, OCT, ophthalmoscopy (indirect ophthalmoscopy), slit lamp exam, fundus photography), confirmation of concomitant medications and medical treatments, IP administration according to PRN dosing criteria, AE evaluation, follow-up contact (for those who had the IP administered)

  * *During the ophthalmological examination, FA and ICGA will be conducted during Visit 12 (9 months) if necessary, at the investigator's discretion.*

⑨Visit 15 (End of Study [EOS]/Early Termination [ET]): 12 months

- Vital signs (blood pressure, pulse, body temperature), pregnancy test (if necessary, only for women of childbearing potential), ophthalmic examination (BCVA, IOP measurement, OCT, FA, ICGA (conduct the tests if deemed necessary by the investigator), ophthalmoscopy (indirect ophthalmoscopy), slit lamp exam, fundus photography), immunogenicity test, laboratory tests (general blood test, blood chemistry test, urine test), confirmation of concomitant medications and medical treatments, AE evaluation

11. Endpoints

➢ Primary efficacy endpoint

Proportion of subjects who lost <15 letters in BCVA score from baseline at 3 months

➢ Secondary efficacy endpoints

- Mean change from baseline in BCVA score at 3, 6, and 12 months
- Proportion of subjects who lost <15 letters in BCVA score from baseline at 6 and 12 months
- Proportion of subjects who gained ≥15 letters in BCVA score from baseline at 3, 6, and 12 months
- Mean change from baseline in CRT by OCT at 1, 3, 6, and 12 months
- Proportion of subjects with no intra-retinal or sub-retinal fluid at 3, 6, and 12 months after administration

➢ Safety

AEs, AEs in the study eye and fellow eye, AEs of special interest (AESIs), physical examination, vital signs, and laboratory tests, etc.

➢ Immunogenicity

Incidence of anti-drug antibody (ADA) and neutralizing antibody due to CKD-701 and Lucentis® at screening, 1, 2, 3, 6, and 12 months

➢ Pharmacokinetics

PK blood samples are collected from 24 people (12 people per group), and the blood sampling time points and endpoints are:

- Blood sampling schedule: pre-dose (Day 0), Day 1 (C_1), Day 7 (C_{w1}), Day 30 ($C_{trough1}$), Day 60 ($C_{trough2}$), and Day 90 ($C_{trough3}$)
- Endpoints: A systemic exposure assessment of CKD-701 and Lucentis® at C_1, C_{w1}, $C_{trough1}$, $C_{trough2}$ and $C_{trough3}$

12. Statistical Analysis

➢ Pharmacokinetics Analyses

PK analysis data are analyzed on the PK analysis set.

For the PK evaluation of ranibizumab, plasma concentration is calculated for each subject and presented using descriptive statistics (mean, standard deviations, etc.) by treatment group.

However, a separate additional analysis may be considered for subjects whose blood is drawn later than 24 hours at C_1.

➢ Efficacy Analyses

In consideration of “Bias toward the null” where treatment effects may be underestimated due to low compliance with the study and thus coincidentally demonstrate equivalence, the primary analysis set is set as the per-protocol set (PPS) and the secondary analysis set is set as the full analysis set (FAS) to analyze the efficacy data.

As for the proportion of subjects who lost <15 letters in BCVA score from baseline at 3 months (the primary efficacy endpoint), the number and the proportion (%) of subjects for each treatment group are presented and Cochran–Mantel–Haenszel test is performed, which adjusts for the presence or absence of baseline polypoidal choroidal vasculopathy (PCV) as a covariate. If the 95% confidence interval (CI) for the difference between the treatment groups is within the equivalence margin of -11.5% to 11.5%, it is determined that the equivalence has been demonstrated.

The secondary efficacy endpoints are presented using the descriptive statistics for the treatment groups at each time point. For continuous variables, the groups are compared using analysis of covariance (ANCOVA), which adjusts for baseline PCV as the covariate, and for categorical variables, using the Cochran–Mantel–Haenszel test, which adjusts for baseline PCV as the covariate.

If necessary, the analysis is carried out for exploratory evaluation purposes for items that may affect medicinal efficacy, and multiplicity adjustments are not made as it is not a confirmatory test result.

➢ Safety Analysis

All safety data are analyzed on the safety analysis set.

All AEs collected during the study are coded using the latest version of Medical Dictionary for Regulatory Activities (MedDRA) (version 20.1 or higher). A treatment emergent adverse event (TEAE) refers to the case where a new AE occurs or symptoms worsen following the first IP administration. AE analysis for safety assessment is based on the TEAEs. The results of AE analysis are presented with the number of subjects and incidence (%) by system organ class (SOC) and preferred

term (PT) for each treatment group, and compared between the groups using Pearson's chi-square test or Fisher's exact test depending on the expected frequency. AEs are classified into AEs, adverse drug reactions (ADRs), serious AEs (SAEs), serious ADRs (SADRs), AEs by severity, and AESIs and analyzed in the same manner, and if necessary, may be presented as a data listing by number of subjects with an event.

In addition, TEAEs are classified into ocular AEs of the study eye, ocular AEs of the fellow eye, and non-ocular AEs and summarized by treatment group, and underlying symptoms that were displayed prior to the first IP administration are presented as data listing for each subject.

For the laboratory test results, the number and proportion (%) of subjects who were assessed as clinically significant abnormal at baseline, 6 months, and 12 months are presented. Any changes in before and after conditions are compared using the McNemar test and inter-group comparisons are made using the Pearson's chi-square test or Fisher's exact test depending on the expected frequency.

Descriptive statistics are presented for the vital signs and physical measurement results at each time point. Intra-group changes are examined via a paired t-test or Wilcoxon signed-rank test, and inter-group comparisons are made via an independent samples t-test or Wilcoxon rank-sum test, depending on the normality test.

The immunogenicity test results are analyzed on the safety analysis set. The immunogenicity test results at each time point are summarized by treatment group and compared between the groups using the Pearson's chi-square test or Fisher's exact test depending on the expected frequency. The immunogenicity may be presented in a data listing by incidence, if necessary.

13. References

1) Bressler SB, Bressler NM, Fine SL, et al. Natural course of choroidal neovascular membranes within the foveal avascular zone in senile macular degeneration. Am J Ophthalmol 1982;93:157-63.
2) Park SJ, Kwon KE, Choi NK, et al. Prevalence and incidence of exudative age-related macular degeneration in South Korea: a nationwide population-based study. Ophthalmology 2015;122:2063-70.e1.
3) Friedman DS, O'Colmain BJ, Muñoz B, et al. Prevalence of age-related macular degeneration in the United States. Arch Ophthalmol 2004;122:564-72.
4) Au Eong KG. Age-related macular degeneration: an emerging challenge for eye care and public health professionals in the Asia Pacific region. Ann Acad Med Singapore 2006;35
5) Park KH, Song SJ, Lee WK, et al. The results of nation-wide regis-try of age-related macular degeneration in Korea. J Korean Ophthalmol Soc 2010;51:516-23
6) Ranibizumab: Phase III Clinical Trial Results Philip J. Rosenfeld, MD, PhD*, Ryan M. Rich, MD,Geeta A. Lalwani, MD Ophthalmol Clin N Am 19 (2006) 361–372
7) Brown DM, Kaiser PK, Michels M, et al. Ranibizumab versus verteporfin for neovascular age-related macular degeneration. N Engl J Med 2006;355:1432-44.
8) Rosenfeld PJ, Brown DM, Heier JS, et al. Ranibizumab for neovascular age-related macular degeneration. N Engl J Med 2006;355:1419-31.
9) Kang S, Cho WK, Roh YJ. The efficacy of ranibizumab for choroidal neovascularization in age-related macular degeneration. J Korean Ophthalmol Soc 2009;50:725-30.
10) Senger, DR; Galli, SJ; Dvorak, AM; Perruzzi, CA; Harvey, VS; Dvorak, HF (25 February 1983). "Tumor cells secrete a vascular permeability factor that promotes accumulation of ascites fluid.". Science. 219 (4587): 983–5.
11) Ferrara N, Henzel WJ. Pituitary follicular cells secrete a novel heparin-binding growth factor specific for vascular endothe-lial cells. Biochem Biophys Res Commun 1989;161:851-858.
12) Vascular endothelial growth factor and age-related macular degeneration: from

basic science to therapy Nature Medicine 16, 1107–1111 (2010).

13) Selection and Analysis of an Optimized Anti-VEGF Antibody: Crystal Structure of an Affinity-matured Fab in Complex with Antigen. J. Mol. Biol. (1999) 293, 865-881.

14) Bacterial host strains, EP 1 341 899 B9.

15) Ranibizumab FDA Statistical Review.

16) Kodjikian L, Souied EH, Mimoun G, et al. Ranibizumab versus Bevacizumab for Neovascular Age-related Macular Degeneration: Results from the GEFAL Noninferiority Randomized Trial. Ophthalmology 2013;120:2300-2309.

17) Holz FG, Amoaku W, Donate J, et al. Safety and Efficacy of a Flexible Dosing Regimen of Ranibizumab in Neovascular Age-Related Macular Degeneration: The SUSTAIN Study. Ophthalmology 2011;118:663–671.

18) Busbee BG, Ho AC, Brown DM, et al. Twelve-Month Efficacy and Safety of 0.5 mg or 2.0 mg Ranibizumab in Patients with Subfoveal Neovascular Age-related Macular Degeneration. Ophthalmology 2013;120:1046–1056.

19) David M. Brown, Peter K. Kaiser, et al. Ranibizumab versus Verteporfin for Neovascular Age-Related Macular Degeneration. The New England Journal of Medicine 2006;355:1432-44.

20) Philip J. Rosenfeld, David M. Brown, et al. Ranibizumab for Neovascular Age-Related Macular Degeneration. The New England Journal of Medicine 2006;355:1419-31.

21) Carl D. Regillo, David M. Brown, et al. Randomized, Double-Masked, Sham-Controlled Trial of Ranibizumab for Neovascular Age-related Macular Degeneration: PIER Study Year 1. American Journal of Ophthalmology 2008;145:239–248.

22) Prema Abraham, Huibin Yue, et al. Randomized, Double-Masked, Sham-Controlled Trial of Ranibizumab for Neovascular Age-Related Macular Degeneration: PIER Study Year 2. American Journal of Ophthalmology 2010;150:315–324.
