# Supplementary material for: Efficacy and safety of a new ranibizumab biosimilar CKD-701 using a pro re nata treatment regimen in neovascular age-related macular degeneration: A phase 3 randomized clinical trial
Source: PLoS One. 2022 Nov 14;17(11):e0275611. doi: 10.1371/journal.pone.0275611 (PMC9662729; doi:10.1371/journal.pone.0275611)
Supplement: Attachment — Submitted filename: Response to Reviewers.docx.doc [file pone.0275611.s004.doc]

The responses to all comments have been prepared and attached herewith.

When submitting your revision, we need you to address these additional requirements.

1. Please ensure that your manuscript meets PLOS ONE's style requirements, including those for file naming. The PLOS ONE style templates can be found at

Response: We confirmed that the manuscript is written in PLOS ONE style template.

2. Thank you for submitting your clinical trial to PLOS ONE and for providing the name of the registry and the registration number. The information in the registry entry suggests that your trial was registered after patient recruitment began. PLOS ONE strongly encourages authors to register all trials before recruiting the first participant in a study.

As per the journal’s editorial policy, please include in the Methods section of your paper:

a) your reasons for your delay in registering this study (after enrolment of participants started);

b) confirmation that all related trials are registered by stating: “The authors confirm that all ongoing and related trials for this drug/intervention are registered”.

Response: We added the reason for delay of clinical trial registration and the stating about registration of related drug in the manuscript. Thank you for your comment. (Page 5 line 86)

3. We note that the original protocol file you uploaded contains a confidentiality notice indicating that the protocol may not be shared publicly or be published. Please note, however, that the PLOS Editorial Policy requires that the original protocol be published alongside your manuscript in the event of acceptance. Please note that should your paper be accepted, all content including the protocol will be published under the Creative Commons Attribution (CC BY) 4.0 license, which means that it will be freely available online, and any third party is permitted to access, download, copy, distribute, and use these materials in any way, even commercially, with proper attribution.

Therefore, we ask that you please seek permission from the study sponsor or body imposing the restriction on sharing this document to publish this protocol under CC BY 4.0 if your work is accepted. We kindly ask that you upload a formal statement signed by an institutional representative clarifying whether you will be able to comply with this policy. Additionally, please upload a clean copy of the protocol with the confidentiality notice (and any copyrighted institutional logos or signatures) removed.

Response: We got the permission from the study sponsor and submitted clean copy of protocol. (S1 file)

4. We note that the original protocol that you have uploaded as a Supporting Information file contains an institutional logo. As this logo is likely copyrighted, we ask that you please remove it from this file and upload an updated version upon resubmission.

Response: We submitted the clean copy of protocol without company logo and confidentiality notice.

5. Thank you for stating the following financial disclosure:

“The study was funded by the sponsor, CKD. Ltd (Chong Kun Dang). Study design, collection and analysis of data were performed by the respective groups of the sponsor.

Korean Association of Retinal Degeneration also funded this study and participated as image reading center (Seoul Reading Center).”

Please state what role the funders took in the study. If the funders had no role, please state: ""The funders had no role in study design, data collection and analysis, decision to publish, or preparation of the manuscript.""

If this statement is not correct you must amend it as needed.

Please include this amended Role of Funder statement in your cover letter; we will change the online submission form on your behalf.

Response: Thank you for your comment. The funders had no role in study design, data collection and analysis, decision to publish, or preparation of the manuscript. We added this statement in the financial disclosure section.

6. In your Data Availability statement, you have not specified where the minimal data set underlying the results described in your manuscript can be found. PLOS defines a study's minimal data set as the underlying data used to reach the conclusions drawn in the manuscript and any additional data required to replicate the reported study findings in their entirety. All PLOS journals require that the minimal data set be made fully available. For more information about our data policy, please see http://journals.plos.org/plosone/s/data-availability.

Upon re-submitting your revised manuscript, please upload your study’s minimal underlying data set as either Supporting Information files or to a stable, public repository and include the relevant URLs, DOIs, or accession numbers within your revised cover letter. For a list of acceptable repositories, please see http://journals.plos.org/plosone/s/data-availability#loc-recommended-repositories. Any potentially identifying patient information must be fully anonymized.

Important: If there are ethical or legal restrictions to sharing your data publicly, please explain these restrictions in detail. Please see our guidelines for more information on what we consider unacceptable restrictions to publicly sharing data: http://journals.plos.org/plosone/s/data-availability#loc-unacceptable-data-access-restrictions. Note that it is not acceptable for the authors to be the sole named individuals responsible for ensuring data access.

We will update your Data Availability statement to reflect the information you provide in your cover letter.

Response: We prepared minimal data set used to reach the conclusion and uploaded this data. (S2 file)

7. Please include captions for your Supporting Information files at the end of your manuscript, and update any in-text citations to match accordingly. Please see our Supporting Information guidelines for more information: http://journals.plos.org/plosone/s/supporting-information.

Response: We added captions for supporting information as your suggestion.

Reviewer #1: Thank you for submitting this interesting work to PLOS ONE.

This is overall a well-designed study on investigating the efficacy, safety and immunogenicity of the CKD-701 biosimilar and a nicely written manuscript.

Here are my comments and suggestions.

1. In the Methods section you mention the “key exclusion criteria”. Are they any additional ones that were either applied to the study eye or the fellow eye such as presence of other concurrent non-retinal pathologies? Also, any systemic diseases or medications? If so, I would highly suggest you include those as well (supplemental material).

Response: We appreciate your review and comments. We excluded patients having ocular disease other than AMD, systemic infection and taking systemic medication which can result in retinal adverse events. Detailed inclusion and exclusion criteria are described in supplementary protocol. (Page 5 line 99 and Supporting file (S1 file) page 4)

2. Regarding the PRN injection criteria, I would recommend you comment on whether all of these or any combination or even only one criterium could be met in order to proceed with the injection.

Response: Clinical trial drug was re-administered when any one of the PRN administration criteria were met. We corrected the PRN treatment protocol in the manuscript clearly. (Page 6 line 124)

3. Was angiography (FA or ICGA) also performed at other points or only at baseline?

Response: Fluorescein angiography was also performed at the end of the study.

4. What about the patients who lost >15 letters?

Response: We added this data, who lost more than 15 letters, in the Table 2.

5. Did you perform a subanalysis on the fellow eyes?

Response: Thank you for your comment. We are preparing the post hoc analysis about this study data including fellow eye characteristics in next publication.

Reviewer #2:

1. Abstract: line 23. The 3 months compared to baseline - when is baseline relative to the initial 3-month loading intraocular injections, and when is the 3 months? Is it before the PRN dosing? This is a little unclear at the moment.

Response: I appreciate your comment. We agree with your opinion that the description is unclear. The secondary outcome time point was 3 months after the baseline visit, one month after last loading injection and before PRN dosing. We corrected the description more clearly. (Page 2 28)

2. lines 28, 30, 32, 34, 181, 186, etc: p-values need only be to 2 decimal places at this value.

Response: We corrected the p-values to be 2 decimal. Thank you for your comment

3. Introduction: line 46: what is "wet" AMD?

Response: Thank you for your comment. We replaced ‘wet AMD’ to neovascular AMD.

4. line 66 - no need for a "," after "Although, ".

Response: We deleted the comma. Thank you for kind comment.

5.

Material and methods

The primary endpoint is whether the patient lost fewer than 15 letters of visual acuity at 3 months compared to the baseline value.

I presume this is the quantity used in the sample size justification in line 137-139, but that doesn't seem to be the situation. What is actually the rate of 30% difference between the ranibizumab and placebo group? It is not currently clear why this sample size was determined - perhaps the rates in the groups as well as the difference?

(is there some confusion with participants who lost<15 letters and participants who gain>= 15 letters?)

Response: I appreciate your comment. In pivotal study of Ranibizumab for wetAMD, MARINA study, proportion of patients who lost fewer than 15 letters were 94.6% in 0.5 mg ranibizumab and 62.2% in sham injection at 12 months. Difference of proportion was more than 30% in this pivotal study. Our primary outcome was measured at 3 months because drug response is most sensitive at this time and the improvement of vision maintained through 1 year. We corrected the reference as MARINA study from ANCHOR study.(Reference 15, Page 7 line 149)

6. line 192: after first administration?

Response: Thank you for your comment. The expression, ‘after first administration’, means after the first intravitreal injection because the first injection was performed on the day of randomization. We corrected this description not to make confusion. (Page 10 line 203)

7. table 2,3,4: p-values needn't be to 4 decimal places. 2 would be satisfactory.

Response: We corrected the p-values to 2-decimal value.

8. Figures refer to the drug as Lucentis - this is not in the text anywhere - ensure there is no ambiguity as to whether these figures are using the same treatment as the text in the study.

Thank you for your comment. I understand your confusion. Pharmacokinetics sample was performed in 12 patients of each CKD-701 (study drug) and Ranibizumab (reference drug). We replaced Lucentis to Ranibizumab to clarify.

9. Was any consideration given to fitting mixed models to the data to account for the differing time points among the same patients in the models? This could provide an average treatment effect for the difference between treatment arms over time - rather than just the 3 month after study entry timepoint.

In general the time points could refer to time since randomisation to reduce uncertainty regarding when the time point was.

Response: Thank you for your comment. We agree with you. We added the description regarding the baseline time point. (Page 6 line 129)
